# Risk factors for proximal junctional kyphosis after posterior long-segment internal fixation for chronic symptomatic osteoporotic thoracolumbar fractures with kyphosis

**DOI:** 10.1186/s12893-022-01598-9

**Published:** 2022-05-14

**Authors:** Qing-Da Li, Jun-Song Yang, Bao-Rong He, Tuan-Jiang Liu, Lin Gao, Xin Chai, Xin Tian, Ding-Jun Hao

**Affiliations:** Department of Spine Surgery, Honghui Hospital, Xi’an Jiaotong University, No.555, Youyi East Road, Xi’an, Shaanxi China

**Keywords:** Elderly patients, Osteoporosis, Spinal fractures, Postoperative complications, Proximal junctional kyphosis, Kyphosis, Internal fixation

## Abstract

**Background:**

This study aimed to analyze the risk factors for proximal junctional kyphosis (PJK) for patients with chronic symptomatic osteoporotic thoracolumbar fractures (CSOTLF) and kyphosis who underwent long-segment internal fixation.

**Methods:**

We retrospectively reviewed the records of patients with CSOTLF complicated with kyphosis who underwent posterior multilevel internal fixation in our hospital between January 2013 and January 2020. The patients’ age, sex, body mass index (BMI), bone mineral density (BMD), smoking status, cause of injury, comorbidities, injury segments, and American Spinal Injury Association (ASIA) grading non-surgical data; posterior ligament complex (PLC) injury, upper and lower instrumented vertebral position (UIV and LIV, respectively), number of fixed segments surgical data, proximal junctional angle (PJA), sagittal vertebral axis (SVA), pelvic incidence (PI), lumbar lordosis (LL), pelvic incidence-lumbar lordosis mismatch (PI-LL), pelvic tilt (PT), and sacral slope (SS) surgical indicators were collected. Patients were divided into postoperative PJK and non-PJK groups.

**Results:**

This study included 90 patients; among them, 30 (31.58%) developed PJK postoperatively. All patients were followed up for > 24 months (mean 32.5 months). Univariate analysis showed significant differences in age, BMI, BMD, PLC injury, UIV, and LIV fixation position, number of fixation stages, and preoperative PJA, SVA, PI-LL, and SS between the two groups (P < 0.05). Additionally, no significant differences were observed in sex, smoking, cause of injury, complications, injury segment ASIA grade, and preoperative PT between the two groups (P > 0.05). Multifactorial logistic regression analysis showed that age > 70 years (OR = 32.279, P < 0.05), BMI > 28 kg/m^2^ (OR = 7.876, P < 0.05), BMD T value < − 3.5 SD (OR = 20.836, P < 0.05), PLC injury (OR = 13.981, P < 0.05), and preoperative PI-LL > 20° (OR = 13.301, P < 0.05) were risk factors for PJK after posterior long-segment internal fixation in elderly patients with CSOTLF complicated with kyphosis.

**Conclusion:**

CSOTLF patients undergoing posterior long segment internal fixation are prone to PJK, and age > 70 years, BMI > 28 kg/m2, BMD T value < − 3.5 SD, preoperative PI-LL > 20° and PLC injury may increase their risk.

## Background

The increasing age of the population has highlighted osteoporosis and associated fractures as major health concerns [1]. However, elderly patients are unresponsive to pain and so injuries often go unnoticed for longer, leading to the progression of acute fractures to chronic symptomatic osteoporotic thoracolumbar fractures (CSOTLF) [2]. Increased patient age, osteoporosis, and progressive vertebral collapse lead to progressive aggravation of thoracolumbar kyphosis, which severely affects the quality of life of patients. However, bed rest, wearing braces, anti-osteoporosis medications, and other non-surgical treatments are ineffective for some patients. Long-segment internal fixation is effective in stabilizing the spine, correcting deformities, reestablishing spinal balance, and relieving neuro spinal compression; however, patients with older age, poor bone conditions, or medical comorbidities are at an increased risk of surgical complications [3]. One common complication of long-segment internal fixation of the spine is the proximal junctional kyphosis (PJK), whose progression can cause intractable low back pain, severe cosmetic deformity, and neurological compression symptoms [4], which may require revision surgery [5]. Postoperative PJK is defined being present when the postoperative angle between the lower endplate of the upper instrumented vertebra (UIV) and the upper endplate of UIV + 2 ≥ 10° and it is > 10° greater than the preoperative angle [6]. The incidence of PJK in elderly patients with spinal deformity after orthopedic surgery is 15–40% [7–9]. However, no studies have identified the risk factors associated with the development of PJK after posterior long-segment internal fixation for patients with CSOTLF, and PJK is associated with a complex pathogenesis involving both surgical and non-surgical factors. This retrospective study aimed to determine the risk factors and prevention methods of PJK in patients who underwent recent long-segment internal fixation. Additionally, it aimed to provide clinical suggestions regarding the prevention and reduction of postoperative PJK.

## Materials and methods

### Inclusion criteria

This study included patients with the following characteristics: (1) definitive diagnosis of single-segment CSOTLF (Cobb > 30° on both flexion radiographs and sagittal CT in the supine position) [10]; (2) collapse of the posterior wall of the vertebral body protruding into the first ≤ 1/3 of the spinal canal; (3) treatment with posterior long-segment (≥ 5 motor segments) internal fixation; (4) ineffective conservative management for > 3 months, including the administration of non-steroidal anti-inflammatory painkillers and anti-osteoporosis drugs; (5) age ≥ 60 years; and (6) bone mineral density (BMD) T-value ≤ − 2.5SD.

### Exclusion criteria

Patients with the following characteristics were excluded: (1) history of thoracolumbar spine surgery; (2) comorbid idiopathic or congenital spinal deformity; (3) history of severe spinal cord injury; (4) pathological fractures caused by tumors, infections, tuberculosis, and other causes; (5) history of lower extremity surgery, such as the knee and hip, which may affect the measurement of imaging data; and (6) < 24 months of postoperative follow-up or incomplete imaging data.

### General data

A total of 136 patients diagnosed with T_11_-L_2_ CSOTLF were treated with posterior long-segment internal fixation between January 2013 and January 2020; 41 patients were excluded based on the exclusion criteria. A total of 95 patients met the inclusion criteria, including 32 males and 63 females; The subjects’ ages ranged from 60 to 85 years (mean: 67.3 ± 6.5 years). The patients were divided into the PJK group (n = 30) and the non-PJK group (n = 65), according to the postoperative development of PJK. The full-length frontal and lateral radiographs of the spine were collected preoperatively, before discharge, 6 months postoperatively and during the final follow-up, which should clearly show the C_7_-L_5_ vertebrae, the sacral plateau and the bilateral femoral head, and the relevant parameters were measured by using Surgimap software. Patients’ age, sex, body mass index (BMI), bone mineral density (BMD), smoking, combined diseases, ASIA grading, follow-up time and fixed segments were recorded. The patients’ demographic details are presented in Table 1. The retrospective study was approved by the Medical Ethics Committee of the Honghui Hospital Affiliated to Xi’an Jiaotong University.

**Table 1:** Demographic data

Groups	Sex		Age	BMD T value	Injury		Level				Time from injury to surgery
	Male	Female	(years)	(SD)	sprain	fall	T_11_	T_12_	L_1_	L_2_	(years)
PJK Group (n = 30)	12	18	73.6 ± 5.3	− 4.5 ± 0.4	6	24	5	12	10	3	5.8 ± 2.2
Non-PJK Group (n = 65)	20	45	64.5 ± 5.2	− 3.5 ± 0.4	25	40	12	25	20	8	6.1 ± 2.8
χ^2^/t value	0.783		7.858	12.164	0.038		0.187				0.396
P value	0.376		0.000	0.000	0.845		0.980				0.652

*PJK* proximal junctional kyphosis, *BMD* bone mineral density

### Surgical techniques

The procedures were performed by the same group of experienced physicians. The patient was placed in the prone position after general anesthesia, and a sponge pad was placed under the chest and pelvis to suspend the abdomen. After routine disinfection and towel laying, a posterior median incision was made at the apex of the posterior convexity of the spine, and the paravertebral muscles were separated to reveal at least two normal vertebral bodies above and below the injured vertebra. The required vertebral segments and upper and lower articular processes were exposed, and pedicle screws were placed in each of the two upper and lower vertebrae of the injured spine. In patients with severe osteoporosis, the fixed segments were extended and the nail tract was reinforced, including one or two sets of screws proximally and distally or selectively depending on the intraoperative situation. After nail placement, single-segment pedicle subtraction osteotomy (PSO) was performed, and the spinous processes, laminae, and bilateral pedicles were removed using biting forceps. Short rods were used to temporarily fix the adjacent segments above and below the osteotomy,and the C-arm X-ray machine was used to confirm the effect of kyphosis correction. After checking that there was no active bleeding, the wound was flushed, a drainage tube was placed, and the surgical incision was closed layer by layer.

### Observation indicator

**Fig. 1:**
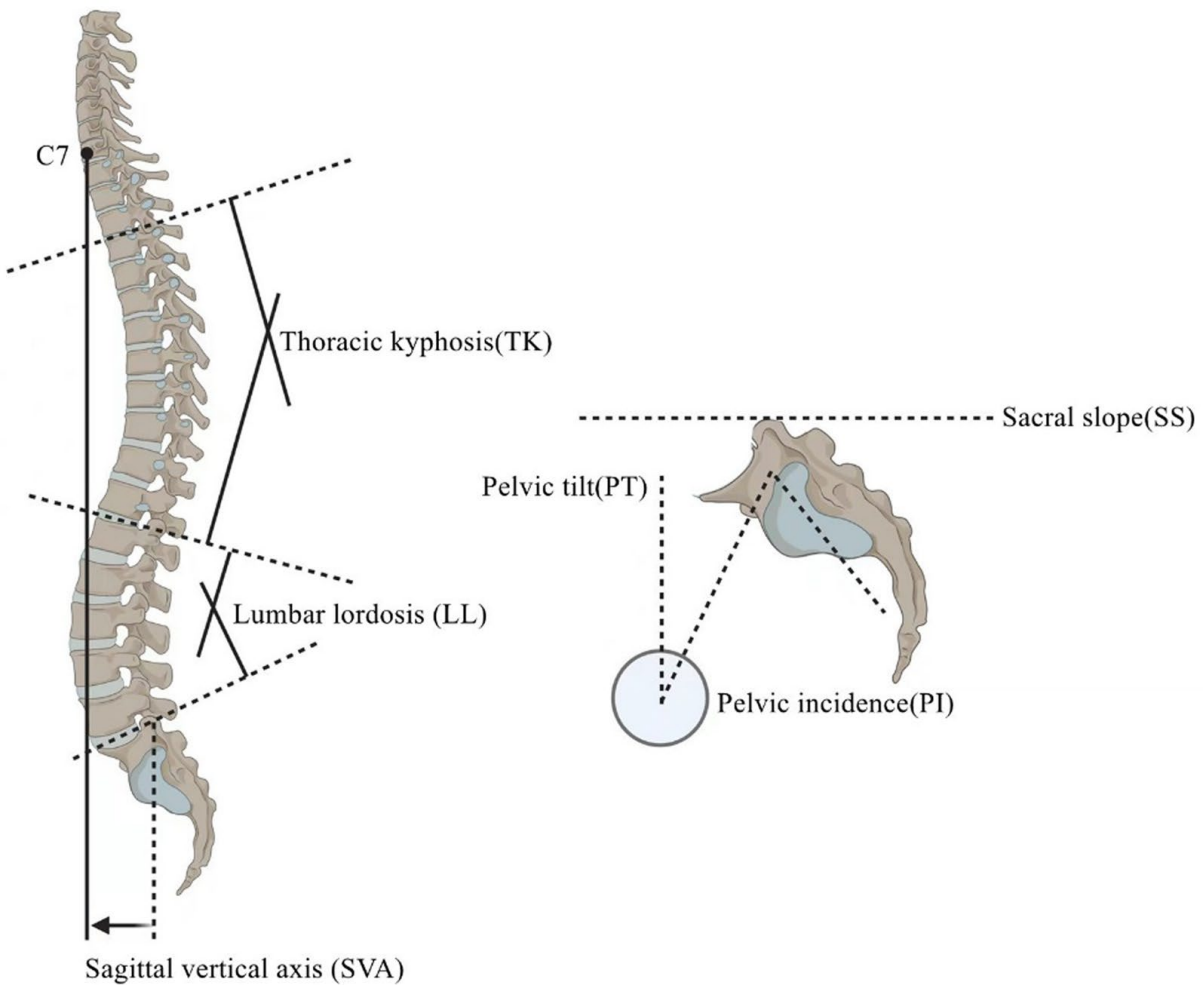
Illustration of various spine pelvic parameters. *SVA* sagittal vertical axis, *PI* pelvic incidence, *LL* lumbar lordosis, *TK* Thoracickyphosis, *PT* pelvic tilt, *SS* sacral slope

(1) The PJA (the angle between the upper endplate of the UIV + 2 and the lower endplate of the UIV); (2) sagittal vertebral axis (SVA) (the vertical distance between the plumb line at the center of the C_7_ vertebral body and the posterior edge of the supra-sacral endplate); (3) pelvic incidence-lumbar lordosis (PI-LL) corresponding to the compatibility between the lumbar curve and pelvic morphology (PI is the angle between the line from the center of the femoral head to the center of the superior sacral endplate and the vertical line of the superior sacral endplate; on the other hand, LL is the angle between the parallel line formed by the T_12_ superior endplate and the S_1_ superior endplate); (4) pelvic tilt (PT) is the angle between the straight line and the plumb line at the end of the superior sacral endplate and the midpoint of the central line of the bilateral femoral heads; (5) sacral slope (SS) is the angle between the S_1_ upper-end plate and horizontal line; (6) PLC injury is a partial or complete tear of a single or joint of the supraspinous ligament, interspinous ligament, or ligamentum flavum, with or without a small joint fracture. (7)UIV position is divided into T_10_ or more and T_10_ to T_12_; (8) lower instrumented vertebral (LIV) position is divided into L_5_ or more and S_1_ (Fig. 1).

### Statistical analysis

All statistical analyses were performed using SPSS software (version 23.0; IBM Corp, Armonk, NY, USA). Continuous data were expressed as *mean* ± *SD*, which were analyzed using independent samples *t-test*. Categorical data were expressed as frequency (percentage), which were analyzed using the chi-square test or Fisher’s exact probability method. Univariate analysis was used to identify potential factors influencing PJK after posterior long-segment internal fixation. Statistically significant variables in the univariate analysis were further analyzed using multivariate logistic regression analysis. P < 0.05 was considered statistically significant, and P < 0.01 was deemed highly significant.

## Results

### The incidence of PJK

In our group of CSOTLF patients, PJK occurred in 30 cases after long-segment internal fixation, with an incidence of 31.6%. In the PJK group, 12 patients had symptoms of low back pain and 3 patients had symptoms of nerve damage in the lower extremities. Among them, 8 patients underwent revision surgery, 3 patients with UIV/UIV + 1 fracture, 2 patients with loose UIV internal fixation, and 3 patients with nerve symptoms in the lower extremities. The patients with nerve symptoms were decompressed by extending the fixed segment 2 ~ 3 segments proximally and ensuring that it was not located in the posterior convex apex of the thoracic spine.

### Univariate analysis

The results of the univariate analysis are shown in Table 2. No significant differences were observed in the sex, smoking history, injury cause, comorbidity, injured segments, ASIA grading, and pre-PT, between the two groups. The proportion of patients aged > 70 was significantly higher in the PJK group (66.7%) than in the non-PJK group (10.8%; χ^2^ = 34.257, P < 0.0001). The proportion of patients with BMI > 28 was significantly higher in the PJK group (70.0%) than in the non-PJK group (12.3%; χ^2^ = 33.035, P < 0.001). The percentage of patients with BMD < 3.5 SD was significantly higher in the PJK group (76.7%) than in the non-PJK group (21.5%; χ^2^ = 26.235, P < 0.0001). The percentage of patients with PLC injury in the PJK group (76.7%) was significantly higher than in the non-PJK group (23.1%; χ^2^ = 24.562, P < 0.001). The percentage of patients whose UIV was positioned at T_10_-T_12_ was significantly higher in the PJK group (70.0%) than in the non-PJK group (35.4%; χ^2^ = 9.982, P = 0.002). The percentage of patients with LIV positioned at S_1_ was significantly higher in the PJK group (63.3%) than in the non-PJK group (36.9%; χ^2^ = 5.779, P = 0.016). The percentage of patients with fixed segments > 7 in the PJK group (63.3%) was higher than that in the non-PJK group (40.0%; χ^2^ = 4.483, P = 0.034). The percentage of patients with pre-PJA > 5° was significantly higher in the PJK group (43.3%) than in the non-PJK group (23.1%; χ^2^ = 4.052, P = 0.044). The percentage of patients with Pre-SVA > 50 mm in the PJK group (60.0%) was higher than that in the non-PJK group (33.8%; χ^2^ = 5.760, P = 0.016). The percentage of patients with PI-LL > 20° was significantly higher in the PJK group (70.0%) than in the non-PJK group (26.2%; χ^2^ = 16.442, P < 0.001), and the percentage of patients with Pre-SS < 25° was significantly higher in the PJK group (56.7%) than in the non-PJK group (32.3%; χ^2^ = 2.526, P = 0.112).

**Table 2:** Univariate analysis of risk factors for PJK

Factors	All patients (n = 95)	PJK Group (n = 30)	Non-PJK Group (n = 65)	χ^2^ value	P value
Age (%)					
60 ~ 64 years	28 (29.5)	1 (3.3)	27 (41.5)	34.257	0.000
65 ~ 70 years	40 (42.1)	9 (30.0)	31 (47.7)		
> 70 years	27 (28.4)	20 (66.7)	7 (10.8)		
BMI (%)					
< 24.0 kg/m^2^	27 (28.4)	2 (6.7)	25 (38.5)	33.035	0.000
24.0 ~ 28.0 kg/m^2^	39 (41.1)	7 (23.3)	32 (49.2)		
> 28.0 kg/m^2^	29 (30.5)	21 (70.0)	8 (12.3)		
BMD (%)					
− 3.5SD ≤ T value ≤ -2.5SD	58 (61.1)	7 (23.3)	51 (78.5)	26.235	0.000
T value < − 3.5SD	37 (38.9)	23 (76.7)	14 (21.5)		
Smoking (%)					
No	62 (65.3)	20 (66.7)	42 (64.6)	0.038	0.845
Yes	33 (34.7)	10 (33.3)	23 (35.4)		
Combined diseases (%)					
Hypertension	35 (36.8)	10 (33.3)	25 (38.5)	0.232	0.630
Diabetes	42 (44.2)	14 (46.7)	28 (66.7)	0.107	0.743
Heart diseases	23 (24.2)	8 (26.7)	15 (23.1)	0.144	0.704
Strokes	26 (27.4)	9 (30.0)	17 (26.2)	0.153	0.696
ASIA grading (%)					
D	19 (20.0)	8 (26.7)	11 (16.9)	1.218	0.270
E	76 (80.0)	22 (73.3)	54 (83.1)		
PLC injury (%)					
No	57 (60.0)	7 (23.3)	50 (76.9)	24.562	0.000
Yes	38 (40.0)	23 (76.7)	15 (23.1)		
UIV (%)					
T_10_ or higher	51 (52.6)	9 (30.0)	42 (64.6)	9.892	0.002
T_10_-T_12_	44 (47.4)	21 (70.0)	23 (35.4)		
LIV (%)					
L_5_ or higher	52 (54.7)	11 (36.7)	41 (63.1)	5.779	0.016
S_1_	43 (45.3)	19 (63.3)	24 (36.9)		
Fixed segments (%)					
≤ 7	50 (52.6)	11 (36.7)	39 (60.0)	4.483	0.034
> 7	45 (47.4)	19 (63.3)	26 (40.0)		
Pre-PJA (%)					
≤ 5°	67 (70.5)	17 (56.7)	50 (76.9)	4.052	0.044
> 5°	28 (29.5)	13 (43.3)	15 (23.1)		
Pre-SVA (%)					
≤ 50 mm	55 (57.9)	12 (40.0)	43 (66.2)	5.760	0.016
> 50 mm	40 (42.1)	18 (60.0)	22 (33.8)		
Pre-PI-LL (%)					
≤ 20°	57 (60.0)	9 (30.0)	48 (73.8)	16.442	0.000
> 20°	38 (40.0)	21 (70.0)	17 (26.2)		
Pre-PT (%)					
≤ 30°	26 (27.4)	5 (16.7)	21 (32.3)	2.526	0.112
> 30°	69 (72.6)	25 (83.3)	44 (67.7)		
Pre-SS (%)					
≤ 25°	38 (40.0)	17 (56.7)	21 (32.3)	5.075	0.024
> 25°	57 (60.0)	13 (43.3)	44 (67.7)		

*PJK* proximal junctional kyphosis, *BMI* body mass index, *BMD* bone mineral density, *ASIA* American Spinal Injury Association, *PLC* posterior ligament complex, *UIV* upper instrumented vertebral, *LIV* lower instrumented vertebral, *Pre* preoperative, *PJA* proximal junctional angle, *SVA* sagittal vertebral axis, *PI* pelvic incidence, *LL* lumbar lordosis, *PI-LL* pelvic incidence-lumbar lordosis mismatch, *PT* pelvic tilt, *SS* sacral slope

### Multivariate analysis

Among the variables that were positive in the univariate analysis, the independent risk factors that were positively correlated with PJK after posterior long-segment internal fixation for CSOTLF were as follows: age > 70 years (odds ratio [OR] = 32.279, 95% confidence interval [CI] = 3.827–272.291, P = 0.001), BMI > 28 kg/m^2^ (OR = 7.876, 95% CI = 1.633–37.993, P = 0.010), BMD T-value < − 3.5SD (OR = 20.836, 95% CI = 2.360–183.928, P = 0.006), PLC injury (OR = 13.981, 95%CI = 1.373–142.344, P = 0.026), and PI-LL > 20°(OR = 13.301,95% CI = 1.544–113.869, P = 0.018). Table 3 show the results of the multivariate analysis.

**Table 3:** Multivariate analysis of risk factors for PJK

Variable	β	SE	Wald	OR value	95%CI	P value
Age > 70 years	3.474	1.088	10.198	32.279	3.827–272.291	0.001
BMI > 28 kg/m^2^	2.064	0.803	6.608	7.876	1.633–37.993	0.010
BMD T value < -3.5SD	3.037	1.111	7.469	20.836	2.360–183.928	0.006
PLC injury	2.638	1.184	4.963	13.981	1.373–142.344	0.026
PI-LL > 20°	2.588	1.096	5.580	13.301	1.544–113.869	0.018

*PJK* proximal junctional kyphosis, *BMI* body mass index, *BMD* bone mineral density, *PLC* posterior ligament complex, *PI-LL* pelvic incidence-lumbar lordosis mismatch, *OR* odds ratio, *CI* confidence interval

## Discussion

In elderly patients, the diagnosis and treatment of CSOTLF are often delayed. Additionally, increasing age leads to worsening of the degree of osteoporosis and thoracolumbar kyphosis; these factors lead to intractable low back pain and eventual bilateral lower extremity paralysis, which severely affect the quality of life of patients [10]. Non-surgical treatment methods are often unsuccessful; however, long-segment internal fixation is effective in maintaining spinal stability, correcting posterior convexity deformity, and preventing injured spine collapse and progression of the posterior convexity Cobb angle [11]. Despite this, long-segment internal fixation can cause several segmental complications, the most common being PJK which affects 6–40% of patients depending on disease type, surgical approach, and follow-up time [12–14]. PJK evaluation criteria are often different for patients with advanced age and excessive deformities [15]. Studies have emphasized that advanced age, BMI, BMD, UIV/LIV fixed position, PJA, LL, SVA, and fixed segments are the main risk factors for developing PJK [16–18]. Clinical observation shows that most patients with PJK develop mild symptoms, requiring only regular check-ups but the condition of some patients may progress to PJF or even neurological damage, which can severely affect patients' postoperative functional recovery. Therefore, when using posterior long-segment internal fixation for CSOLTF, active detection of high-risk factors for PJK is important for improving patient prognosis.

### Univariate analysis of possible factors for PJK

The results of univariate analysis in this study showed that the risk factors associated with the occurrence of postoperative PJK in patients included age, BMI and BMD:

1. Age. Advanced age is a risk factor for developing PJK. Particularly, previous studies found that individuals aged > 55 years have the highest incidence of PJK, and the risk increases with age [19]. Kim et al. [20] and Yang et al. [21] also confirmed that PJK is more prevalent and has a higher incidence in people aged ≥ 60 years. Patients with PJK can be aggravated by spinal deformities, which leads to changes in paravertebral muscle tissue; furthermore, uneven stresses on the intervertebral discs and small spinal joints can accelerate degeneration. In addition, advanced age is considered to be an important risk factor for revision surgery for PJK [13].
2. BMI. Currently, patients with BMI > 25 kg/m^2^ are believed to have high susceptibility to PJK. This relationship may be attributed to the increased load placed on the spine and internal fixation in overweight and obese patients and the forward shift of the body’s center of gravity, resulting in increased stress on adjacent segments. Simultaneously, obese and overweight patients have significantly weakened low back muscles, which increases the risk of osteoporosis [22].
3. BMD. O'Leary et al. [23] showed that patients with osteoporosis are more prone to PJK. Additionally, reduced bone mass and bone microarchitecture disruption reduce screw holding power, which increases the risk of screw loosening and extraction. In addition, lower bone density is associated with reduced muscle tissue in the thoracolumbar segment, which may lead to spinal instability and accelerate the development of PJK [24].

Preoperative spine imaging data plays an important role in evaluating spinal balance. The results of univariate analysis in this study showed that preoperative PJA, preoperative SVA and preoperative PI-LL were associated with postoperative PJK:

1. PJA and PJK. A retrospective study by Annis et al. [25] showed that a postoperative PJA > 5° was correlated with the occurrence of postoperative PJK. The author concluded that the greater the preoperative PJA, the greater the degree of surgical orthosis and more damage to the proximal junctional area and posterior ligamentous structures, resulting in biomechanical changes in the junctional area leading to the occurrence of PJK.
2. SVA and PJK. Iyer et al. [26] found that the SVA increases with age, and when the sagittal sequence is artificially corrected, the organism still has the tendency to return to its natural state. Furthermore, overcorrection of the retro-convex deformity increases the stress in the proximal junction area and increases the incidence of PJK.
3. PI-LL and PJK. PI-LL reflects whether the lumbar curve is compatible with the morphology of the pelvis and indicates the compensatory state of the sagittal balance of the spine. Senteler et al. [27] found that higher PI-LL increased the compression and shear forces in the L_3_, L_4_, and L_5_ motion segments, leading to accelerated degeneration of the adjacent segments, thus increasing the risk of PJK. Therefore, it is important to adequately measure and analyze spinal imaging data before surgery and to intervene in high-risk groups by identifying risk factors to reduce the occurrence of PJK after surgery.

Surgery may have altered the local anatomy and biomechanics of the spine. The results of the univariate analysis in this study showed that PLC injury status, UIV position, LIV position and number of fixed segments correlated with the occurrence of postoperative PJK:

1. PLC: Posterior spinal surgery may damage the proximal soft tissues, including the supraspinous and interosseous ligaments; furthermore, small joint capsule injuries can lead to decreased local stability and PJK [28].
2. UIV/LIV: Additionally, the choice of UIV/LIV is related to the occurrence of PJK; therefore, the apex of posterior convexity and segments with degenerative instability should be avoided as much as possible. UIV fixation to the thoracolumbar segment increases the risk of PJK, which may be attributed to the fixation of the UIV at the transition from thoracic kyphosis to the anterior lumbar convexity. This area is the junctional zone of stress transmission and is relatively less stable due to the lack of thoracic protection, which increases the susceptibly for PJK. When the UIV is fixed above T_10_, stable rib support exists, which can protect the stability of the adjacent vertebrae and reduce the occurrence of degenerative diseases in the proximal adjacent segments. However, this can cause increased intraoperative bleeding; furthermore, no significant advantages have been found regarding other complications and revision rates. The literature states that LIV fixation fusion to the sacrum/pelvis/iliac bone is twice as effective as preserving lumbosacral motion for PJK [29]. The author believes that fusion to S_1_ results in a concentration of proximal stresses due to long segment fixation, a weakened sagittal balance of pelvic regulation, and a higher incidence of both postoperative pseudarthrosis and sagittal imbalance. Adult spinal deformities often have structural changes and deformities at the L_4/5_ segment; therefore, fixation at L_5_ avoids iliac screws, preserves lumbosacral motion, and reduces sacroiliac joint stresses. However, extended fixation to the ilium can effectively increase fixation strength and stability in the following patients: patients with coronal/sagittal imbalance of the trunk who require orthopedics; those who require three-column osteotomy in the lumbosacral region; those with repeated nail placement or poor stability of S_1_ screws found intraoperatively; and those with significant lumbosacral instability or revision surgery. However, it should be noted that the choice of UIV/LIV is not absolute fixation; particularly, the patient’s spinal balance should be considered in the decision.
3. Fixed segments: Internal fixation with long segmental pedicle screws increases the incidence of PJK since it can damage more paravertebral muscles and small intervertebral joints while exposing the nail placement point, which affects spinal stability and stress, especially at the proximal junction, the junction that generates lateral stress and causes displacement of the adjacent vertebrae. Kim et al. [8] found that patients were at an increased risk for PJK if the number of fixed vertebral bodies was > 5. This may be attributed to the significant concentration of stress in the adjacent vertebral body, making the adjacent vertebral body more susceptible to degeneration and displacement.

### Multivariate analysis of risk factors for PJK

The results of multifactorial logistic regression analysis in this study showed that age > 70 years, BMI > 28 kg/m^2^, BMD T value < -3.5 SD, preoperative PI-LL > 20° and PLC injury possibly increased risk of postoperative PJK after posterior long-segment internal fixation. In elderly CSOTLF patients with the above risk factors, attention should be paid to adjusting the patient's general comprehensive condition to reduce the incidence of PJK.

The patient's own factors mainly include:

1. Functional exercise of the lumbar back muscles: As patients age, paravertebral muscle atrophy and severe paravertebral fat infiltration lead to decreased paravertebral muscle strength, which significantly increases the risk of postoperative PJK [30]. Therefore, appropriate preoperative muscle exercises can help reduce the occurrence of PJK.
2. Weight reduction: Reducing a patient’s weight can lower the physical stress of the musculoskeletal system in the proximal junction area, which reduces the incidence of PJK.
3. Anti-osteoporosis treatment: Standardized anti-osteoporosis treatment can improve bone calcium content and bone strength, which can help maintain the stability of the internal fixation system and reduce loosening and extraction. Therefore, it can reduce the development of vertebral fractures and collapse in the junction area, which reduce the incidence of mechanical complications.

Imaging related factors are mainly sagittal sequence reconstruction:

Sagittal restoration: moderate deformity correction while considering overall balance should be performed according to the adult spinal deformity sagittal evaluation standard SRS-Schwab staging requirements [31] and spinal sagittal sequence score (global alignment and proportion score) [32].

Surgery-related factors include:

1. Soft tissue protection: Anatomical exposure of the upper end of the vertebral region minimizes the damage to the supra- and interspinous ligaments. Fine separation of the paravertebral muscles in the junctional area, maximum possible preservation of the midline ligament structure, and muscle attachment [33].
2. Ligamentous strengthening of the junctional area: ligamentous strengthening through tendon grafting or silk wire reinforcement can reduce the stress in the junctional area and increase the strength of the PLC [34].
3. Non-strength fixation: the use of a plate hook in the proximal fixed vertebrae can provide a relatively non-strength fixation structure, which can help protect the small joints and discs of the adjacent segments, prevent excessive stress concentration in the junctional zone, and reduce the occurrence of PJK or PJF [35].
4. UIV/LIV selection: in the apex of the lordosis and the segment of degenerative disability, proximal and distal fixed vertebrae should be avoided; on the other hand, proximal fixed vertebrae should be avoided in the thoracolumbar junction area. Regarding distal fixed vertebrae, advantages and disadvantages of fixation in L_5_, S_1_, or the skeleton should be considered on an individual basis [36].
5. Bone cement reinforcement: preventive application of bone cement reinforcement to both fixed and adjacent vertebrae to increase the strength of the vertebral body can, to a certain extent, avoid internal fixation failure and adjacent vertebrae fracture [37].

### Limitations

This was a single-center retrospective study with a small number of enrolled patients and selection bias. Most of the relevant clinical evaluation information during the follow-up period was provided by the patient through telephone interview or outpatient follow-up, which may have caused errors. Future multicenter prospective studies with large sample sizes are warranted to understand the relationship of PJK with different imaging parameters, and fixed segments. This is to guide the surgical strategy of long-segment fixed fusion, minimize the risk of postoperative PJK, and provide guidance on the strategy of revision surgery.

## Conclusion

Elderly patients with CSOTLF and kyphosis aged > 70 years with BMI > 28 kg/m^2^, BMD < 3.5 SD, preoperative PI-LL > 20°, and PLC injury appear to have an increased risk of PJK after posterior long-segment internal fixation. It is suggested that for elderly patients with CSOLTF, a detailed preoperative evaluation should be performed to develop a reasonable surgical plan to avoid a greater degree of kyphosis correction. Intraoperative soft tissue protection, preservation of PLC, and UIV/LIV avoidance in the thoracolumbar segment and sacrum should be emphasized. For patients with significant sagittal imbalance, targeted measures should be administered preoperatively; furthermore, postoperative follow-up and testing should be increased to emphasize the postoperative recovery of sagittal balance and reduce the risk of PJK.

## Data Availability

The datasets used and/or analysed during the current study are available from the corresponding author on reasonable request.

## Abbreviations

CSOLTF: Chronic symptomatic osteoporotic thoracolumbar fractures
PJK: Proximal junctional kyphosis
BMI: Body mass index
BMD: Bone mineral density
ASIA: American Spinal Injury Association
PLC: Posterior ligament complex
UIV: Upper instrumented vertebral
LIV: Lower instrumented vertebral
Pre: Preoperative
PJA: Proximal junctional angle
SVA: Sagittal vertebral axis
PI: Pelvic incidence
LL: Lumbar lordosis
PI-LL: Pelvic incidence-lumbar lordosis mismatch
PT: Pelvic tilt
SS: Sacral slope
OR: Odds ratio
CI: Confidence interval
